# Population-based cancer registries in Nigeria and the National Cancer Control Programme

**DOI:** 10.3332/ecancer.2023.1592

**Published:** 2023-08-25

**Authors:** Ismail Hadi Zubairu, Muhammad Shakir Balogun

**Affiliations:** 1 Department of Radiology and Oncology, College of Medical Sciences, Ahmadu Bello University, Zaria 810107, Nigeria; 2 Nigeria Field Epidemiology and Laboratory Training Programme, African Field Epidemiology Network, 50 Haile Selassie Street, Asokoro, Abuja 900231, Nigeria; a https://orcid.org/0000-0002-3661-3250; b https://orcid.org/0000-0002-4846-9698

**Keywords:** cancer registries, registries, sub-Saharan Africa, Nigeria, developed countries, international agencies

## Abstract

Population-based cancer registries (PBCRs) are important sources of data on cancer burden in a defined population. They are a cornerstone for establishing, implementing and monitoring and evaluating a rational cancer control programme. Despite the long history of PBCRs in more developed countries of the world, PBCRs in sub-Saharan Africa are still poorly developed and cancer control is poor. Compared to PBCRs in Europe and the United States, cancer registries in sub-Saharan Africa are still functioning at a basic level. Only a few cancer registries in sub-Saharan Africa contribute data regularly to the International Agency for Cancer Registries’ (IACR) GLOBOCAN and Cancer in Five Continents’ publication series. In Nigeria, there have been efforts at strengthening existing PBCRs and creating new ones, and implementing national cancer control programmes. It is however unclear how successful these efforts have been. It is therefore necessary to reflect on documented activities so far in order to identify gaps and proffer solutions.

## Introduction

A population-based cancer registry (PBCR) is a system that collects information from multiple sources on all reportable malignant neoplasms occurring in a geographically defined population [1]. Population-based cancer registration represents the gold standard for providing information on cancer incidence in a defined population [2]. The PBCR can serve to identify possible causes of cancer in the community and to assess the impact of cancer control activities, and it is a core component of cancer control strategy [2].

The concept of PBCR has been in existence for more than five decades [3]. The first PBCR was founded in the 1930s [3]. Currently, there are more than 700 PBCRs worldwide at varying stages of development [4]. Well-developed PBCRs have gone beyond performing the perfunctory roles of estimating rates and comparing cancer profiles [3]. A PBCR seeks information from multiple sources, chiefly, hospital records and diagnostic departments, including cytopathology, histopathology and medical imaging. Death registries may document cancer as the cause of death and contribute to PBCR data [2]. This makes obvious the relevance of computerised health information systems in storing patients’ medical records. It eases the cumbersome process of linking many different sources of relevant data. It is critical to link together all of the records that concern an individual to avoid duplication. Linking computerised health information systems with PBCRs expands their capacity beyond the traditional registry dataset. For example, details of the treatment a patient received (conventionally, only the type of treatment, e.g., chemotherapy, is recorded in the registry, but not the regimen and doses used, or whether the patient is currently receiving second- or third-line chemotherapy, for example) and the treatment outcome can be documented. Essential variables typically captured in PBCRs include personal identification, age, gender, residential address, ethnicity, date of onset of symptoms, basis of diagnosis, site of primary cancer, histology and source of information [2]. Recommended variables include the stage of disease at diagnosis, initial treatment, date of last contact and status at last contact (at least dead or alive) [2].

Data from PBCRs provide valuable information on the impact of interventions across the cancer control spectrum including prevention, early detection, treatment, rehabilitation and palliation. For example, in 1994, the cancer registry in Taiwan compared the incidence of liver cancer in children between the age of 6 and 9 years, who were born before the introduction of hepatitis B vaccination for newborns and those who were born after. They found a four-fold decrease in incidence following the introduction of vaccination [3]. Similarly, in cancer screening, a study of cervical cancer screening programmes and policies in Europe found a high concentration of organised screening programmes in areas that have long-standing cancer registries [5]. This was attributed to the link between pilot programmes for cervical cancer screening and cancer registration.

The World Health Organisation recommends that as an initial step to setting up a National Cancer Control Programme (NCCP), an analysis of the cancer burden, risk factors in the target area and capacity assessment (analysis of existing facilities, programmes and services) are conducted [2]. This can only be achieved with a cancer surveillance that is built around a PBCR [2]. The PBCR will provide the data needed for a comprehensive assessment of the cancer burden. It is desirable to have incidence, survival and mortality data for all forms of cancer combined and for each of the most common cancers. Other indicators such as person-years, life lost and disability-adjusted life years may also be calculated [2]. These data provide justification for establishing a cancer control programme, as well as monitoring and evaluating the programme. Where PBCRs do not exist, cancer data are estimated based on the evaluation of time trends of incidence or mortality data and variations according to age group, sex and other relevant characteristics [2].

Despite these well-recognised utilities of PBCRs, they are either entirely not available in many developing countries or are poorly developed. It is therefore difficult to accurately determine the cancer burden – in terms of incidence, distribution and survival – in these countries. Without these data, implementing a rational cancer control plan and evaluating it is impossible.

Although Nigeria started the process of developing cancer registries in the 1970s, those efforts have not been very successful [6]. The absence of quality data from Nigeria in the past few decades in recognised cancer data repositories, such as the GLOBOCAN published by the International Agency for Research on Cancer (IARC) and Cancer in Five Continents (CIV), is evidence of the failure of these efforts. According to the IARC classification of cancer registries, Nigeria belongs to category IV, which means that registration activities are hospital- and/or pathology-based systems, rates cannot be calculated or there is no documented evidence of efforts to establish a PBCR [7]. In recent times, however, there have been renewed efforts at strengthening existing cancer registries and establishing new ones. There are many challenges when setting up a PBCR, such as educating stakeholders in the cancer care space about the relevance of PBCRs will help break resistance to adopting additional financial and work responsibilities such as providing the infrastructure necessary to support the PBCR, recording relevant patient data, and sustaining the processes involved in cancer registration to ensure that the goals of the PBCR are achieved. This review highlights the features of PBCRs, the situation of PBCRs in Nigeria, the relevance of PBCRs in cancer control programmes, and comparisons with other PBCRs and cancer control programmes in sub-Saharan Africa. The purpose is to review literature and reports to identify the gaps in Nigeria’s NCCP and to gain insights into other NCCPs in sub-Saharan Africa.

## Methodology

We have conducted a narrative review to allow critical analysis of review articles and published government reports. This approach best suits the aim of our research because of the diverse issues and fields that are being analysed. According to some authors, narrative reviews are effective where the aggregation of data is difficult because different subject themes are being analysed [8].

**Figure 1.**
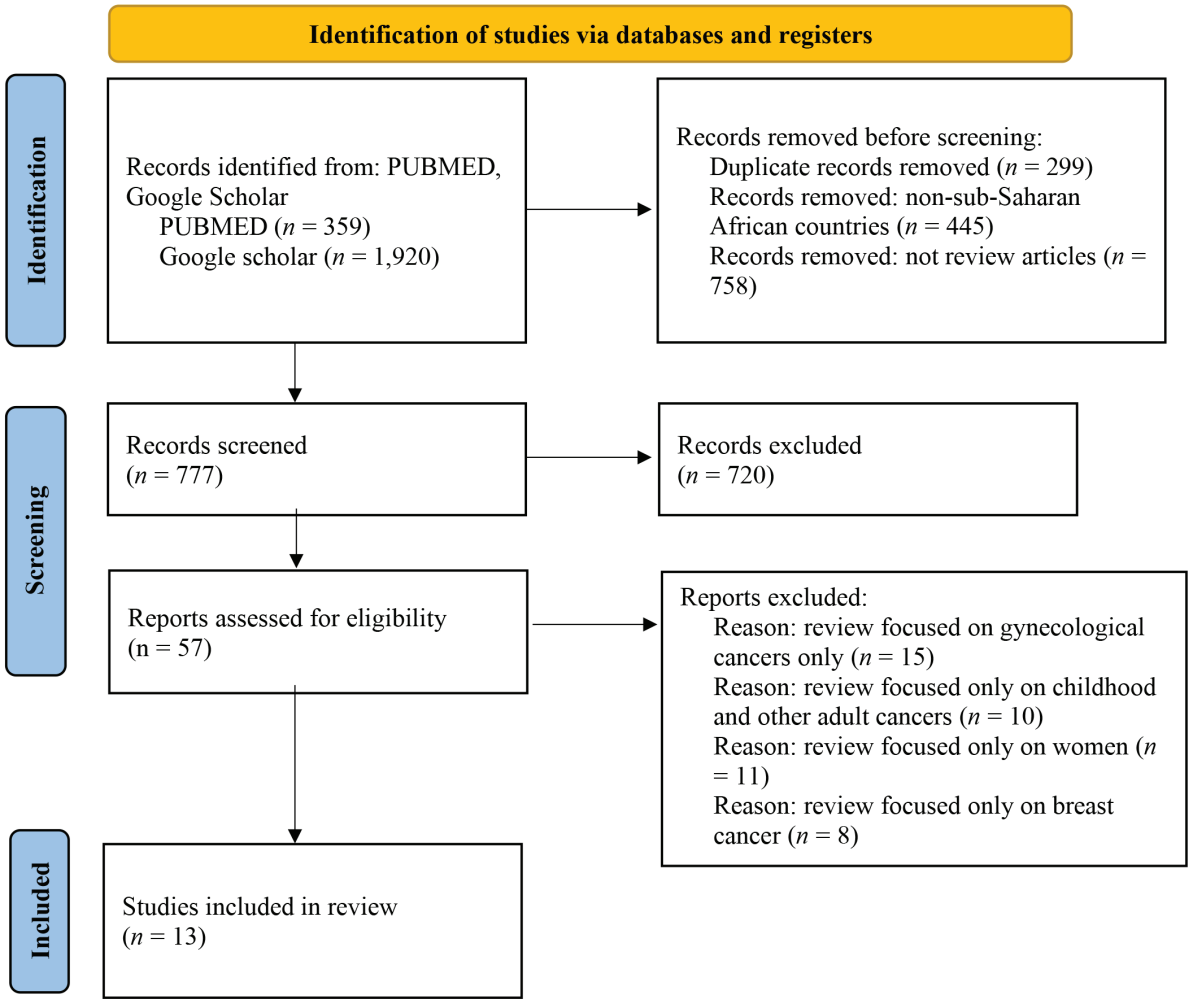
Literature search flow diagram.

A literature search was conducted on PUBMED and Google Scholar using the Query: ‘PBCRs AND NCCP AND sub-Saharan Africa OR low-middle-income countries’. The search on PUBMED yielded 359 results and 1920 on Google Scholar. Duplicate studies were eliminated; studies that included non-African countries, and those that focused on a particular demographic population, e.g., children, and specific cancer sites, e.g., breast cancer, were also excluded. The search was further refined to include only review articles (this is because we wanted to focus on only information that is relevant to the objectives of the study) published from 2005, and in English language. Only articles that focused on the features and dynamics of PBCRs and cancer control were selected. Ten articles were finally included in the review. In addition, a country each was selected from west (Ghana), east (Kenya), and southern Africa (South Africa), and the official policy document of their NCCP was included in the review. We have shown the process of literature search and selection in the flow chart (Figure 1). The inclusion and exclusion criteria are shown in Table 1.

## Discussion

### PBCRs in Nigeria

The Nigerian National Systems of Cancer Registries (NSCR) was established in 2009 as a collaboration between the Federal Ministry of Health (FMOH), the Society of Oncology and Cancer Research of Nigeria and the Institute of Human Virology of Nigeria with the primary objectives of providing training, capacity development, monitoring and providing technical and scientific support to cancer registries in Nigeria. A publication in 2015 reported the activities of this organisation, its achievements, the challenges it encountered, a map for future directions and suggestions for future activities pertaining to strengthening PBCRs [8]. Notable among the achievements of NSCR is the successful submission and publishing of cancer data from PBCR in Abuja, Ibadan and Calabar in the GLOBOCAN 2012 publication that was launched on 12 December 2013 [8]. The data published were the most common cancers documented from this registry and their age-standardised incidence rates (ASR). Data reporting on the ASR from 11 other PBCRs were published in 2 separate peer-reviewed journal articles [8]. Other efforts to submit data to the GLOBOCAN publication have failed because they did not meet up with the requirements for data quality set by the IARC. Similarly, efforts to submit data generated from the Ibadan PBCR to the prestigious IARC publication, *CIV*, failed because they did not meet the criteria set for data quality. However, data from other sub-Saharan African countries, such as Malawi, Zimbabwe, Kenya and Uganda, were included [8].

In the 13 years of its existence, the NSCR has been able to train more than 70 registry directors, cancer registrars and data clerks on basic cancer epidemiology and the use of CanReg5 – a cancer registration software developed by the IARC [8]. In its first 5 years, the NSCR conducted 51 mentoring and monitoring visits to 17 cancer registries all over the country [8]. The NSCR also disseminates information on cancer registration to relevant institutions through newsletters in print and online. The NSCR has a website where information on the number of PBCRs and Hospital-Based Cancer Registries (HBCRs), and the contact details of the directors and registrars for each PBCR are provided. The only publication – *Cancer in Nigeria 2009–2016* – is also available for download. There are a total of six newsletters published, the most recent was in January 2017. The website also hosts data from five cancer registries – Abuja, Edo, Ekiti, Sokoto and Calabar – with application procedures to be able to access these data [9].

The efforts deployed by the NSCR to strengthen PBCR although many and laudable, do not appear to have the coordination and sustainability necessary to cause a bigger impact. For example, the last newsletter published was about 6 years ago, in 2017 [9]. The publication *Cancers in Nigeria* was last published in 2016, and there is no evidence to suggest that the data from contributing cancer registries have been updated recently [10].

### History of cancer registries in Nigeria

**Figure 2.**
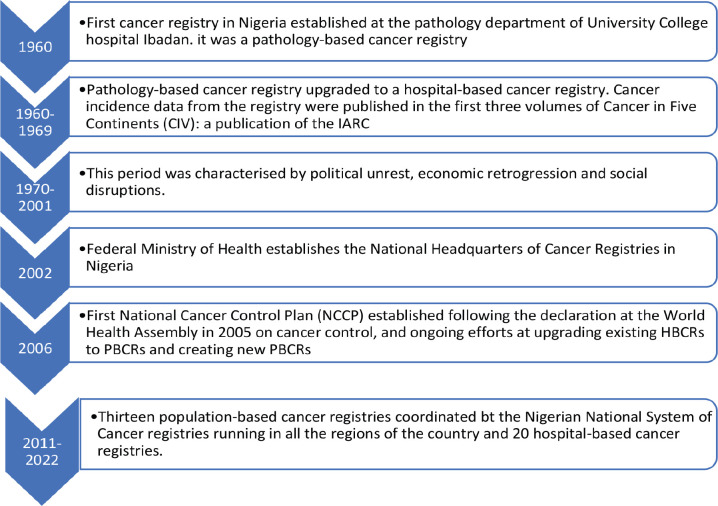
Evolution of cancer registration in Nigeria.

**Figure 3.**
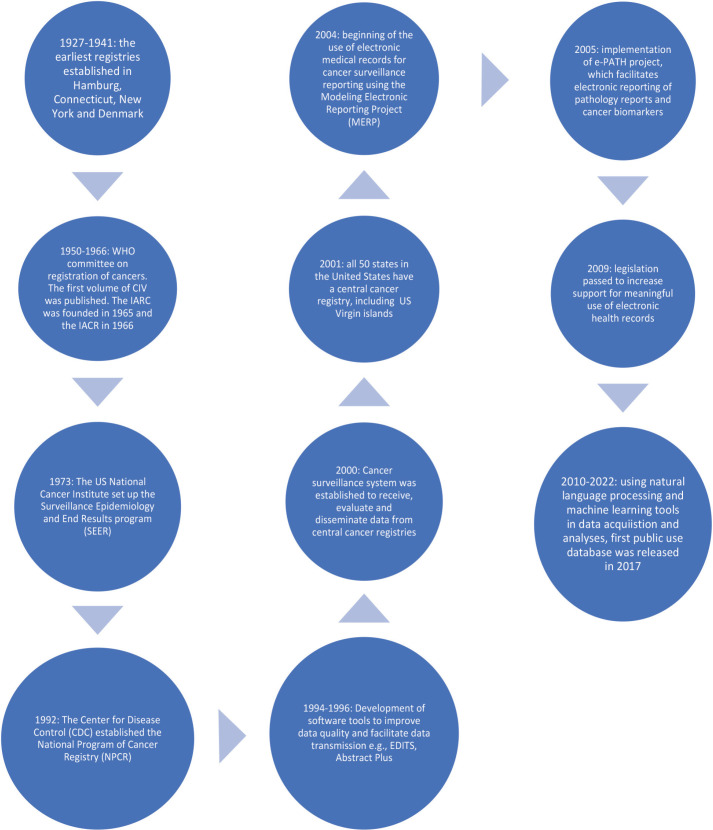
Timeline for the evolution of cancer registries in the world.

The first cancer registry in Nigeria was an HBCR at the Pathology Department of the University College Hospital, Ibadan. It was established in the 1960s [8]. Cancer incidence data from this registry were published in the first three volumes of CIV – 1960–1962, 1960–1965 and 1960–1969 [11]. In the following decades up till 2001, there were no contributions from any cancer registry in Nigeria to CIV or any other global cancer databases [8]. This was attributed to political unrest, economic retrogression and social disruptions. During this time, most published cancer data were obtained from case series and records from pathology departments [12]. Subsequently, there was an attempt to coordinate cancer data collection by the Nigerian FMOH through the establishment of the National Headquarters of Cancer Registries in Nigeria; however, this did not last as the organisation stopped functioning in 2002 following the death of its executive chairman [8]. There was no other effort until around 2008, following the 58th World Health Assembly Resolution on Cancer Prevention and Control adopted in May 2005. Nigeria set up its first NCCP in 2006 and subsequently put efforts at upgrading the existing HBCRs to PBCRs and creating more PBCRs [13]. Figure 2 highlights the evolution of cancer registration in Nigeria, and Figure 3 shows the timeline of the evolution of the functions and roles of PBCR in developed countries.

### Nigerian NCCP

The first NCCP was officially launched in 2008 and was expected to be operational for 5 years, elapsing in 2013 [13]. The second edition was launched in 2018, expected to span 5 years, through 2022 [14]. On 4 November 2022, at the commemoration of the International Cancer Week in Abuja, the third edition of the NCCP was launched. This also has a timeframe of 5 years and is expected to elapse in 2027. The key elements of the NCCP have remained constant since its inception; however, there are some improvements in its operationalisation. For example, the plan for funding the programme was not clearly stated in the first edition. There was also no clear plan on how to ensure patients’ access to quality and equitable cancer care, surveillance of cancer survivors and integration of cancer care into other relevant health programmes. These have been improved upon in the second and third editions. The second edition (2018–2022) drafted a policy that captures elements of the cancer continuum from prevention to palliative care and survivorship. It clearly stipulated a budget of about 300,000 U.S. dollars, 75% of which would be provided by the Nigerian government, and 25% contributed by donors and development partners [14]. The policy document lists the following as its primary goals: 1) accessible screening services for all Nigerians, 2) improved access quality and cost-effective diagnosis and treatment services, 3) improved quality of life, 4) increased awareness and advocacy, 5) cancer data and research, 6) availability and accessibility of relevant medicines and equipment and 7) coordination of resources for cancer care. The anticipated impact was: 1) reduced incidence of common cancers, 2) improved financing from the government and private sector and 3) reduced morbidity and mortality. All the priority areas for intervention, which ultimately lead to achieving a specific goal, were thoroughly interrogated and a plan detailing the activities to undertake, the responsible agency/department/body and key partners, expected outcomes, anticipated risks and risk mitigation strategies, as well as timeframes for completion were clearly outlined. The strategies, key performance indicators and targets for each goal were stated. The anticipated cost for each intervention was also clearly planned for on a year-by-year basis. A major limitation of this programme is the absence of baseline data. Most of the planning is based on calculated estimates. The interventions directed at prevention and screening for example were planned for without an accurate guide on the incidence and prevalence of the priority cancers in the population. A well-coordinated system of PBCRs would have provided the critical information necessary to ensure a more effective and efficient use of scarce resources.

Although the NCCP 2023–2027 has been launched, there is no report yet of the just concluded NCCP 2018–2022. Therefore, it is rather early to assess the success of the programme. It would be interesting to note the baseline surveys conducted to arrive at baseline data upon which the success or otherwise of the interventions can be assessed. It would also be interesting to note how successful the prevention and screening strategies have been, considering that up till now there is no national cancer screening programme for neither cervical cancer, breast cancer, prostate cancer or colorectal cancer. The human papillomavirus vaccination has not been incorporated into the extended programme for immunisation (EPI). The bill on tobacco smoking has been passed and adopted. Out of the eight public comprehensive cancer centres in the country, only three currently have functioning megavoltage radiotherapy machines and rendering services. The others are limited to rendering surgery, chemotherapy and palliative care to cancer patients. There has been a regular supply of syrup morphine for pain control in palliative care patients at all tertiary health facilities offering oncology services. The chemotherapy access programme initiative has been successful in providing chemotherapeutic agents to patients at a subsidised rate. Advocacy by stakeholders in the cancer care space has led to the incorporation of certain monoclonal antibodies used in cancer treatment into the national health insurance where the pharmaceutical company, the National Health Insurance Agency, and the patient share the cost of the medicine.

### Cancer registries and cancer control in sub-Saharan African countries

#### Ghana

Ghana launched its cancer control programme in 2011 and planned to span for 5 years from 2012 to 2016 [15]. The Ghanaian policy document adopted a systematic disease-wise approach where seven priority cancers were identified. General risk factors for cancers, such as obesity, cigarette smoking, alcohol consumption and occupational and environmental exposure, were identified, and specific activities to mitigate these risk factors were clearly stated. For example, legislation regulating public smoking, alcohol consumption and indiscriminate use of pesticides in agricultural produce, integration of human papilloma virus (HPV) vaccination into the EPI and specific steps towards educating and motivating Ghanaians to adopt healthy lifestyles including increased physical activity. The document was clear on training healthcare providers on screening methods, frequency of screening and improving competence in early cancer detection. There are only two health facilities providing comprehensive cancer care in Ghana. It was not clearly stated if these centres are adequate to cater to the oncology needs of the Ghanaian population and what clear steps would be taken to increase the number to meet deficits. However, plans to increase equipment and infrastructure for diagnosis and treatment procedures were mentioned in passing. There was no clear plan for ensuring an adequate and uninterrupted supply of medicines, vaccines and equipment for health facilities offering cancer care. Although a budget summary was presented, it is not clear how this budget was funded. At the time of writing this article, there is no evidence that this programme has been evaluated.

Similar to Nigeria, the history of cancer registries in Ghana is fraught with many failed attempts. The most recent was initiated in 2016, as a part of the NCCP, and aimed at strengthening the process [16]. The project was supported by a grant and technical support from Stanford University. The activities towards setting up the PBCR were grouped into five categories, namely:

1. Policy: a policy document on cancer control mandating cancer registration using the CanReg5 software.
2. Data source: which involved training cancer registrars who actively visit relevant departments to abstract data from the patient medical records.
3. Administrative structure: this includes a registry director, registry manager, an advisory board comprising heads of departments of pathology, oncology, surgery, child health, gynaecology, internal medicine, public health and representatives from birth and death registries, private laboratories and private hospitals.
4. External support and training: which ensures funding, training of staff and technical support.
5. Coverage area: this defines the geographical location covered by the PBCR [16].

The efforts of the PBCR in Ghana have been slightly impactful even if there is still some room for improvement. In a review of cancer incidence published in GLOBOCAN 2012, incidence estimates submitted from Ghana were classified as F, which is the least acceptable quality for inclusion in the analysis [16]. The IARC publication GLOBOCAN accepted national incidence data from the PBCR in 2018 even if it scored low in terms of quality. The data were awarded a score of 8 on a scale of 0 (best) to 10 (worst) [16].

#### Kenya

The first National Cancer Control Strategy (NCCS) in Kenya was launched in 2011–2016. The second edition which was launched in 2017 elapsed in 2022 [17]. The policy is an elaborate document that outlines in detail the strategies to achieve cancer control among Kenyans using five broad pillars. 1. Prevention, early detection and screening, 2. Diagnosis, registration and surveillance, 3. Treatment, palliative care and survivorship, 4. Coordination, partnership and financing, 5. Monitoring, evaluation and research. This strategy was informed by findings from the impact study and derives inputs from several stakeholders. The policy outlines key interventions in a systematic and coordinated manner to implement activities within the cancer control continuum. It clearly outlines the roles of national and county governments, civil society organisations, private sector, academia, healthcare providers and insurance agencies, the public and health development partners, the pharmacy board and cancer registries [17]. There is a clear collaboration between the national and county levels of health care to achieve cancer control. Compared to the Nigerian NCCP, the Kenyan NCCS is more able to succinctly present its goals, objectives and strategies to attain cancer control.

Kenya has a very active PBCR system, the Kenya Medical Research Institute (KEMRI) Cancer Registry [18]. The Nairobi and Eldoret registries are members of the International Agency for Cancer Registries (IACR) and have met the quality standards set by the IARC [19]. Cancer data from these registries are regularly published in the GLOBOCAN series [18].

#### South Africa

South Africa only launched its National Cancer Strategic Framework (NCSF) in 2017 and was scheduled to elapse in 2022 [20]. This cancer control policy document was developed with a vision to provide equitable and comprehensive cancer prevention and control for all South Africans. Previously, there were isolated efforts aimed at addressing some cancer risk factors. However, these efforts were neither coordinated nor collaborative. For example, the Tobacco Products Control Act 83 of 1993, its amendment Act in 1999 and the population-based human papillomavirus vaccination programme started in 2014 [20]. In 2011, the National Cancer Registry (NCR-SA) was established, and the registration of confirmed cancer cases was made mandatory [21]. The Ministerial Advisory Committee on the Prevention and Control of Cancer was established in 2013, and it has played the role of liaison between all the stakeholders in cancer management. In 2017, policies on breast and cervical cancer prevention and management were approved [20]. The NCSF has been designed to leverage these prior achievements to achieve a more effective and efficient cancer control.

The NCSF policy spells out the process of care from surveillance, publicity and education, screening and diagnosis, treatment, supportive care and rehabilitation, patient care pathways, and care packages for individual patients at each level of health care. The document however fails to clearly outline specific activities at each level aimed at clearly defined objectives. The roles of each stakeholder in the cancer care space are also not clear. There are no baseline data against which any improvements can be measured. Although the document identifies common modifiable risk factors, it fails to outline clear activities which will be implemented to mitigate these risk factors. Similarly, for screening, the document listed cancer types that will be prioritised for screening programmes; however, it did not comment on which screening modalities would be deployed.

South Africa currently does not have a PBCR. The NCR-SA is a pathology-based registry established in 1986 [22]. In the decades since its establishment, it has not made much progress in evolving into a PBCR. The NCR-SA is the primary source of cancer data in South Africa; being a pathology-based registry, typically, it will be missing a lot of data concerning clinical features of the disease, including staging, treatment type and treatment outcomes [22]. Furthermore, cancer diagnoses that are made from clinical assessment and medical imaging will also be missing from this data. There are two other cancer registries operating independently in South Africa, the Eastern Province Cape Cancer Registry and the South African Pediatric Tumor Registry [22]. The NCR-SA has a director, quality assurance manager, operations manager and registrars. It employs a total of 17 staff [23]. Data from this registry are not accepted by the IARC [23]. Although efforts are being made to upgrade the existing registry to a PBCR and to establish at least four new sentinel sites per the IACR recommendation, there is no evidence to suggest that this has been achieved [22]. Table 2 compares and contrasts the features of the cancer registries and cancer control programmes in select sub-Saharan African countries.

Although the basic function of PBCRs, i.e., providing data on incidence rates of cancers in a defined geographical location, was the primary focus of the first cancer registries in Europe and America, their roles have since evolved [3]. Well-developed PBCRs now provide information on patterns and causes of cancer, survival from cancer at the population level, and the effects screening, prevention, early diagnosis, treatment and survivorship care have on cancer patients in the population [3]. This information is valuable for research, and to monitor and evaluate cancer control programmes. Cancer registration in more developed countries has evolved significantly from when it first started in the early twentieth century [3]. There are multiple agencies, programmes, and systems that ensure surveillance, data collection and reporting, as well as incorporating technology in the process of cancer registration [25]. Figure 3 highlights the various milestones attained in cancer registration globally.

A synthesis of the literature search shows that PBCRs in Nigeria are still a very long way from achieving most of the successes that PBCRs in more developed countries have achieved. For example, the cancer registries in Nigeria rely on the manual collection of relevant hospital records from pathology departments, surgical outpatient departments, oncology clinics and wards by trained registrars who visit several hospitals within their geographical location periodically. This process is burdensome, inefficient and may result in the collection of poor data. The quality of the data collected may be fraught with errors because of human factors, such as lack of attention to detail as a result of fatigue, large volumes of work, and discontent with remuneration. Furthermore, the data collected using this method are often limited and fail to capture other crucial information that can contribute significantly to providing a more accurate picture of the cancer burden in Nigeria. Compared to the United States, for example, where cancer registries have been linked to electronic medical records since 2004, advanced data collation and analyses using machine learning are being done [25].

Another dimension to look at PBCRs in Nigeria is the number of registries relative to its population. Nigeria is a large country (923,770 km^2^) [26] and the most populous country in Africa, with a population of more than 200 million in 37 administrative states [6]. The geographic size of Nigeria and the size of its population make the establishment of PBCRs that will cover its population, a challenging endeavour. On the Nigerian National System of Cancer Registries’ website, only 9 PBCRs are clearly listed. Three in the South-west, two in the North-west, one in North-central, one in South-east, two in South-south and none in the North-east geopolitical zones of Nigeria. Comparatively, Kenya has a geographical size of 582,646 km^2^ and a population of about 48 million in 47 administrative counties. Kenya has three main PBCRs located in Nairobi, Eldoret and Kisumu counties, and cover less than 10% of the Kenyan population [19]. Nigeria has a landmass almost twice the size of Kenya, and a population about five times that of Kenya. A quick comparison between both countries would show that Nigeria needs about six more PBCRs to equal the ratio between PBCRs and the total population in Kenya.

Apart from the number of PBCRs relative to the population size, a critical aspect is the functionality and effectiveness of the registries. The International Agency for Cancer Registries is an organisation recognised worldwide that collects quality cancer registration data from member organisations and publishes them periodically. It serves to ensure that the data published are of the highest quality based on a set of criteria. Only registries that produce quality data are included. Hence the successful publication of a registry’s data in the GLOBOCAN or CIV can be used as a measure of its functionality and effectiveness. The PBCRs in Nigeria have not successfully published their data in either of GLOBOCAN or CIV in decades. On the other hand, PBCRs in neighbouring Ghana and Kenya have published theirs in recent years.

The poor functioning of the PBCRs in Nigeria will invariably constitute a major setback to the success of its national cancer control plans. The unavailability of accurate data on the burden of cancer on the population makes it difficult to identify key priority areas of intervention and allocate scarce resources to meet immediate needs and plan for the future. It is therefore pertinent that the existing PBCRs be strengthened to function more effectively in order to plan better for cancer control.

## Recommendations and conclusion

PBCRs in Nigeria are not functioning optimally. This will constitute a major setback to the implementation, evaluation and overall success of the NCCP. The Kenyan PBCRs are performing at a higher level and can serve as a model for the Nigerian PBCR given that both countries are low-and-middle income countries (LMICs) and in sub-Saharan Africa. It is critical that PBCRs in Nigeria are revisited and strengthened to be able to provide accurate data on the cancer burden in the country, as well as provide insight into the capacity of the existing service delivery systems to provide quality and equitable care for its citizens.

The NSCR is a veritable tool for coordinating the activities of the existing PBCRs. However, the organization seems to have lost its zeal in the past few years. This may be as a result of inadequate funding by the federal government, lack of political will, and paucity of resources from other sources besides the government. Besides the role of the NSCR, the individual PBCRs must be strengthened by local ownership and investment. This can be achieved by state governments, hospital management and collaborations between public health systems in each geo-political region and non-governmental organisations. Strengthening PBCRs to function optimally will ease the task of the NSCR.

Globally, the activities of PBCRs are coordinated by the IACR. It is therefore important that PBCRs in Nigeria aim to publish their data in the IARC GLOBOCAN publications. This will serve as a critical indicator of the success of our PBCRs and herald the success of our NCCP.

## Conflicts of interest

The author(s) declare that they have no conflict of interest.

## Funding

We have not received any financial support to conduct this study.

## Figures and Tables

**Table 1.** Inclusion and exclusion criteria for review articles included in this study.

Inclusion criteria	Exclusion criteria
Review articles published from 2005 focused on PBCRs. Government reports on policies regarding cancer control in sub-Saharan Africa Review articles focused on NCCP in Nigeria Review articles focused on NCCP in sub-Saharan Africa	Articles that were not in English language. Articles that were limited to only specific types of cancers or specific demographics, e.g., age or gender. Articles that involved non-African countries.

**Table 2.** Comparison between PBCRs and NCCP in select sub-Saharan African countries.

Ghana	South Africa	Kenya
The NCCP in Ghana was initiated in 2011 and expected to span 5 years from 2012 to 2016. In 2012, the Kumasi PBCR was established, and data from this registry was published in the GLOBOCAN publication [24].	The NCSF was launched in 2017 and elapsed in 2022. Previously, efforts at cancer control were isolated and targeted specific disease sites, e.g., cervical cancer and breast cancer. These efforts caused the initiation of the population-based human papillomavirus vaccination programme in 2014, mandatory registration and notification of any cancer, strengthening of the NCR-SA, and ultimately, the NCSF.	The NCCS was first launched in 2011, and spanned for 5 years, up to 2016. The second edition was launched in 2017 and elapsed in 2022. The policy document for this programme is elaborate and clear in its goals and strategies to achieve set goals. There is a clear synergy between the different levels of care, and with the levels of government in executing the cancer control strategy.
Several attempts at establishing PBCRs have failed. The most recent attempt was initiated in 2016 in collaboration with Stanford University, and this has ensured a slow but certain improvement. This has led to the establishment of the Accra cancer registry [16].	The NCR-SA is a pathology-based registry. Efforts are being made to upgrade it to a PBCRs, and to establish at least four more in South Africa. Data from the registries in South Africa are not accepted for publication in GLOBOCAN by the IACR.	Kenya has a very active PBCRs system; the KEMRI. The Nairobi and Eldoret cancer registries are also PBCRs, and all contribute regularly to the GLOBOCAN series.
Data from the cancer registries in Ghana have successfully published cancer incidence in the GLOBOCAN 2012 and 2018 editions.		
